# Metagenomes from Experimental Hydrologic Manipulation of Restored Coastal Plain Wetland Soils (Tyrell County, North Carolina)

**DOI:** 10.1128/MRA.00882-20

**Published:** 2020-10-08

**Authors:** Ariane L. Peralta, Regina B. Bledsoe, Mario E. Muscarella, Marcel Huntemann, Alicia Clum, Brian Foster, Bryce Foster, Simon Roux, Krishnaveni Palaniappan, Neha Varghese, Supratim Mukherjee, T. B. K. Reddy, Chris Daum, Alex Copeland, I-Min A. Chen, Natalia N. Ivanova, Nikos C. Kyrpides, Tijana Glavina del Rio, Emiley A. Eloe-Fadrosh

**Affiliations:** a Department of Biology, East Carolina University, Howell Science Complex, Greenville, North Carolina, USA; b Institute of Arctic Biology and Department of Biology and Wildlife, University of Alaska Fairbanks, Fairbanks, Alaska, USA; c Joint Genome Institute, Lawrence Berkeley National Laboratory, Berkeley, California, USA; University of Southern California

## Abstract

Hydrologic changes modify microbial community structure and ecosystem functions, especially in wetland systems. Here, we present 24 metagenomes from a coastal freshwater wetland experiment in which we manipulated hydrologic conditions and plant presence. These wetland soil metagenomes will deepen our understanding of how hydrology and vegetation influence microbial functional diversity.

## ANNOUNCEMENT

Microbial community structure, soil physicochemical properties, and the abundance and composition of vegetation interact to influence biogeochemical functions (1). Changes in wetland hydrology due to drought, draining, and rewetting cause shifts in soil redox potential, microbial community composition, and associated ecosystem processes such as greenhouse gas (GHG) production (2–6). Vegetation also affects microbial processes by facilitating the transport of oxygen into the root zone and the transport of methane from the root zone into the atmosphere (7, 8). Here, we present wetland soil metagenomes from a mesocosm experiment in which we manipulated hydrology and plant presence to examine microbial community responses. These data complement targeted amplicon sequencing data, GHG fluxes, and soil physicochemical properties from the wetland mesocosm experiment (9).

We collected wetland soils from a restored coastal freshwater wetland at the Timberlake Observatory for Wetland Restoration on the Albemarle Peninsula in Tyrell County, North Carolina (latitude, 35.8959; longitude, −76.1658). We collected soils from three locations with different water table levels (−20 cm, −10 cm, and 0 cm) (10). We altered redox conditions by manipulating hydrology over 8 weeks. The experimental design and sampling details were published by Bledsoe and Peralta (9). We collected and combined six soil cores (3-cm diameter, 10-cm depth) from plant or no-plant areas. We completed 16S rRNA amplicon sequencing (NCBI BioProject PRJNA636184), GHG flux measurements, and soil environmental analyses (9). We sequenced metagenomes that represented the most distinct microbial communities based on amplicon sequencing, and we chose the following samples: wetland soils sampled from field sites at which the water table measured −20 cm and 0 cm, to capture “mesocosm baseline” (*n* = 8) functional diversity, and a subset of samples at the end of the 8-week hydrologic manipulation (i.e., prolonged drying or wetting only) in the presence or absence of vegetation (*n* = 16) (9).

We used the Qiagen DNeasy PowerMax soil kit to extract genomic DNA from freeze-dried soils. Purified DNA products were sent to the U.S. Department of Energy (DOE) Joint Genome Institute (JGI) for sequencing and analysis. Metagenomes were sequenced at the DOE JGI (GitHub [see SupplementalTableS1_MetagenomeSummary_Peralta_et_al.csv at https://doi.org/10.5281/zenodo.4042110]), and project information can be accessed under GOLD (11) study project accession number Gs0142547 and NCBI BioProject accession number PRJNA641216. Plate-based DNA library preparation for Illumina sequencing was performed according to published protocols in GitHub (see Supplemental_Methods_Details_MRA_WetlandSoilMetagenomes.txt at https://doi.org/10.5281/zenodo.4042110). The sequencing project resulted in 583.2 Gbp of raw sequence data. The average read length for each metagenome is found in GitHub (see SupplementalTableS1_MetagenomeSummary_Peralta_et_al.csv at https://doi.org/10.5281/zenodo.4042110). These data were processed using the DOE JGI Metagenome Annotation Pipeline using IMG/M v.5.0.9 (12–14). Initial sequence quality control details can be found in GitHub (see Supplemental_Methods_Details_MRA_WetlandSoilMetagenomes.txt at https://doi.org/10.5281/zenodo.4042110). Illumina reads were quality control filtered according to the protocol described in GitHub (see Supplemental_Methods_Details_MRA_WetlandSoilMetagenomes.txt at https://doi.org/10.5281/zenodo.4042110).

Annotation and gene calling resulted in 600,507 ± 172,049 annotated contigs per sample (mean ± standard deviation [SD]), with a mean of 998,604 gene features (SD, 307,431 gene features) identified in each (Table 1). Based on phylogenetic associations (determined on the basis of bidirectional best hits to genes in other genomes), the estimated alpha diversity across all metagenomes is 10,365 ± 837 operational taxonomic units (OTUs) (mean ± SD) (GitHub [see SupplementalTableS1_MetagenomeSummary_Peralta_et_al.csv at https://doi.org/10.5281/zenodo.4042110]).

**TABLE 1:** Annotation and gene-calling summary for the 24 metagenomes

Gene type and database used^*a*^	No. (mean ± SD)
CDSs	992,951 ± 305,299
rRNAs	775 ± 173
Other genes	4,878 ± 2,340
CDSs with COGs	633,925 ± 194,834
CDSs with SMART	127,732 ± 38,536
CDSs with SUPFAM	641,238 ± 189,914
CDSs with CATH FunFam	509,413 ± 154,168
CDSs with Pfam	603,115 ± 187,921

a CDSs, coding sequences; COGs, Clusters of Orthologous Groups; SUPFAM, superfamily; FunFam, functional families.

### Data availability.

Metagenomes were sequenced at the DOE JGI, and the study information can be found under GOLD study project accession number Gs0142547 and NCBI BioProject accession number PRJNA641216. Additional sample-specific metagenome statistics can be found in GitHub (see SupplementalTableS1_MetagenomeSummary_Peralta_et_al.csv at https://doi.org/10.5281/zenodo.4042110). Details on metagenomic library preparation and sequence filtering can be found in GitHub (see Supplemental_Methods_Details_MRA_WetlandSoilMetagenomes.txt at https://doi.org/10.5281/zenodo.4042110).
